# Chaotic Characteristics Analysis of a Strongly Dissipative Nonlinearly Coupled Chaotic System and Its Application in DNA-Encoded RGB Image Encryption

**DOI:** 10.3390/e28040413

**Published:** 2026-04-04

**Authors:** Zhixin Yu, Zean Tian, Biao Wang, Wei Wang, Ning Pan, Yang Wang, Qian Fang, Xin Zuo, Luxue Yu, Yuxin Jiang, Long Tian, Feiyan Yan

**Affiliations:** 1 College of Mathematics and Big Data, Guizhou Education University, Guiyang 550018, China; yuzhixin@hrbeu.edu.cn (Z.Y.); wangbiao_quirky@126.com (B.W.); wangwei102070@163.com (W.W.); ning_cecile_pan@126.com (N.P.); wangyangsimon@163.com (Y.W.); fqpaper@163.com (Q.F.); zuoxin@gznc.edu.cn (X.Z.); yuluxue2026@163.com (L.Y.); 18486397389@163.com (Y.J.); tianlongah@163.com (L.T.); 15688065653@163.com (F.Y.); 2 College of Big Data and Information Engineering, Guizhou University, Guiyang 550025, China; 3 Guizhou Key Laboratory of Artificial Intelligence and Brain-Inspired Computing, Guizhou Education University, Guiyang 550018, China; 4 College of Computer Science and Electronic Engineering, Hunan University, Changsha 410082, China

**Keywords:** hyperchaotic systems, RGB images, information entropy, image encryption, DNA coding

## Abstract

This paper proposes a novel four-dimensional strongly dissipative nonlinearly coupled hyperchaotic system, investigates its dynamical characteristics, and demonstrates its applicability through Deoxyribonucleic Acid (DNA)-encoded RGB image encryption. First, a four-dimensional nonlinearly coupled hyperchaotic system with strong dissipativity is constructed. Nonlinear dynamics analysis methods, including phase trajectory diagrams, Lyapunov exponent spectra, and bifurcation diagrams, are employed to thoroughly reveal the system’s complex dynamical evolution mechanisms. The analysis indicates that the system not only possesses a wide range of chaotic parameters but also exhibits rich phenomena of multiple coexisting attractors, demonstrating a high degree of multistability. This characteristic offers potential advantages for image encryption, as it increases the diversity of dynamical behaviors and enhances sensitivity to initial conditions. The physical realizability of the chaotic behavior is further verified through an analog circuit implementation. Consequently, the system supports the design of encryption algorithms with larger key spaces, stronger resistance to phase space reconstruction, and improved pseudo-randomness, making it particularly suitable for applications with extremely high security requirements. Subsequently, leveraging the highly random chaotic sequences generated by this system, combined with various DNA coding rules and operations, the RGB image components are scrambled and diffused for encryption. Security analysis demonstrates that the algorithm effectively passes examinations across multiple dimensions, including histogram analysis, information entropy, adjacent pixel correlation, Number of Pixel Change Rate (NPCR), Unified Average Changing Intensity (UACI), and The Peak Signal-to-noise Ratio (PSNR). It achieves favorable encryption results, significantly enhances image resistance against attacks, and provides a reliable technical solution for the secure transmission of remote sensing and military images.

## 1. Introduction

The rapid advancement of remote sensing imaging technology has driven pivotal technological breakthroughs in satellite observation. Satellites can now capture the full spectral range from ultraviolet and visible light to infrared instantaneously and acquire terabyte-level high-resolution images, thus furnishing continuous, multi-scale and highly time-sensitive aerospace information support for meteorological forecasting, disaster early warning, climate change research, national defense reconnaissance and other relevant fields [1,2]. However, high-resolution remote sensing images are highly susceptible to security attacks such as eavesdropping, tampering and forgery during satellite ground transmission, cloud storage and cross-domain sharing [3]. Traditional symmetric and public-key encryption algorithms can hardly meet their security protection requirements. To this end, it is imperative to combine the inherent characteristics of remote sensing data streams, integrate the pseudo-randomness of hyperchaotic systems, the sparse sampling robustness of compressive sensing and the physical noise fingerprints of satellite imaging, and construct a new paradigm for high-resolution remote sensing image encryption with low power consumption, high throughput, quantum resistance and support for real-time on-board operation, so as to meet the urgent demand for remote sensing data security in diverse scenarios including medical emergency response, educational and scientific research, intellectual property protection, and national security [4,5,6].

As a key branch of nonlinear dynamics, chaos theory has been widely applied in the field of remote sensing imaging security due to the complexity and unpredictability of its system behaviors as well as its extreme sensitivity to initial conditions. High-dimensional hyperchaotic systems possess more abundant dynamic evolution characteristics and multi-stability, thus providing a brand-new technical approach for the on-orbit encryption processing of remote sensing images. Consequently, a growing number of studies have applied the characteristics of chaotic systems to the design of encryption algorithms for remote sensing images. In recent years, numerous image encryption schemes based on chaotic mapping have been proposed by scholars, and such encryption technologies have now been extensively adopted [7,8,9,10]. Some scholars have significantly improved the scrambling and diffusion efficiency of hyperspectral remote sensing images by constructing a fusion framework of a five-dimensional hyperchaotic system and an optical neural network [11,12,13]; others have embedded absolute-value memristors into a four-dimensional hyperchaotic system to develop a parallel compressive sensing encryption framework suitable for remote sensing images [14,15,16]. In addition, certain cryptographic systems are developed by fusing multiple chaotic systems, and chaotic cryptography technology has also been effectively applied in the encryption of remote sensing images [17,18]. Researchers have combined quantum long short-term memory (QLSTM) networks [19], three-level radial diffusion, transformation techniques and other methods with chaotic cryptography technology, further enhancing the security of the encryption system. Wang et al. proposed a quantum image encryption algorithm based on the improved Lorenz chaotic sequence of the quantum long short-term memory (QLSTM) network [20]. This algorithm takes the ultra-high-dimensional and unpredictable chaotic sequence generated by QLSTM as the pseudo-random data source to drive the three-level radial diffusion and generalized Arnold-W transformation under the quantum NCQI model, realizing high-intensity scrambling and diffusion of the gray values and positions of quantum images, thereby improving the encryption security of single chaotic systems. Other researchers have adopted technologies such as compressive sensing and combined them with chaotic system environments to achieve the integrated compression and encryption of remote sensing images. Du Yang et al. proposed an optical image encryption algorithm based on a four-dimensional memristor hyperchaotic system (4D-MHS) and compressive sensing [21]. This algorithm takes the complex sequence with high dimensionality and a large key space, output by the newly constructed memristor hyperchaotic system, as a pseudo-random number generator to drive the compressive sensing measurement matrix and the entire scrambling-diffusion process, which significantly improves the brute-force attack resistance and image reconstruction quality of the algorithm.

In this study, a novel four-dimensional strongly dissipative nonlinearly coupled hyperchaotic system is proposed and systematically investigated. The dynamical properties are analyzed through phase portraits, Lyapunov exponent spectra, bifurcation diagrams, and multistability characteristics, and physical realizability is further validated through circuit implementation. The generated chaotic sequences are subsequently employed to construct a DNA-encoded RGB image encryption scheme. By combining chaotic scrambling, diffusion operations, and hash-assisted key generation, the proposed encryption framework achieves strong security and robustness against common attacks. The main contribution of this work lies in the construction and analysis of the new hyperchaotic system, whereas RGB image encryption serves as an application example demonstrating its practical effectiveness.

## 2. 4D System Description and Characteristic Analysis

### 2.1. System Model

The classical Lü system is a three-dimensional autonomous chaotic system [22]. This system is dissipative and extremely sensitive to initial conditions and parameters; furthermore, it can be extended to a hyperchaotic system without equilibrium points. All attractors are hidden and coexist in multiple states, making it suitable for image encryption and secure communication. The mathematical model is given by:

(1) $$\left\{\begin{array}{l} \dot{x}=a(y-x)\\ \dot{y}=-xz+cy\\ \dot{z}=xy-bz \end{array}\right.$$

Based on the advantages of the above-mentioned system, a new four-dimensional hyperchaotic system is proposed by introducing a new state variable *w*:

(2) $$\left\{\begin{array}{l} \overset{•}{x}=a^{2}y+z\omega \\ \overset{•}{y}=x-b\omega \\ \overset{•}{z}=c-xy-z\\ \overset{•}{\omega }=y^{3}-\omega \end{array}\right.$$

In *a* = 1.6, *b* = 2.25, *c* = 1.2, initial value (*x*(0), *y*(0), *z*(0), *w*(0)) = (2, 0, 0, 0) system in state of chaos.

Several four-dimensional hyperchaotic systems derived from the classical Lü system have been reported in the literature. Representative examples include the system proposed by Pang and Liu [23], which extends the Lü system to four dimensions by introducing a linear controller while preserving the original linear structure a(*y* − *x*). Another example is the fractional-order hyperchaotic Lorenz system employed by Wang et al. [24] for image encryption, where the system parameters and derivative order are embedded to enhance security; however, their focus is on application rather than reconstruction of the dynamical structure itself. More recently, Ding et al. [25] proposed a four-dimensional hyperchaotic system for local encryption of vector map data, emphasizing spatial hierarchical indexing and parallel encryption strategies.

While these systems generate hyperchaos by introducing an additional feedback state or nonlinear controller, they largely retain the original Lü-family dynamical framework—particularly the linear structure a(*y* − *x*) and relatively simple coupling mechanisms. In contrast, the present model does not simply append a fourth state to the original Lü equations; instead, it fundamentally reconstructs the coupling structure. This is achieved by replacing the linear term a(*y* − *x*) with *a*^2^*y*, introducing nonlinear cross-coupling through *zω*, and incorporating a cubic nonlinearity *y*^3^ in the new variable’s dynamics. These modifications result in stronger nonlinear interactions and more complex dynamical behavior. Therefore, while inspired by the classical Lü system, the proposed system represents a distinct direction in Lü-type evolution—shifting from linear extension to nonlinear reconstruction—and is more appropriately viewed as a Lü-inspired four-dimensional hyperchaotic system rather than a direct extension.

Compared with existing four-dimensional chaotic systems, the proposed system in Equation (2) introduces a new coupling structure that enhances its dynamical complexity. The originality of this system lies in three aspects: First, the term *zω* in the first equation establishes a multiplicative coupling between the state variables *z* and *ω*, which is rarely observed in conventional 4D systems that typically employ linear coupling. Second, the fourth equation introduces a cubic nonlinearity *y*^3^, which intensifies the nonlinear characteristics and facilitates the generation of hyperchaotic behavior. Third, unlike most systems that rely on simple linear feedback, the proposed system incorporates nonlinear interactions among multiple variables, leading to richer dynamical behaviors such as multiple scroll attractors and higher Lyapunov exponents. To the best of our knowledge, such a combination of nonlinear terms and coupling structure has not been reported in the previous literature, thereby establishing the originality of Equation (2).

#### 2.1.1. Fixed-Point Analysis

Accordingly, the fixed points satisfy:

(3) $$\left\{\begin{array}{l} a^{2}y+z\omega =0\\ x-b\omega =0\\ c-xy-z=0\\ y^{3}-\omega =0 \end{array}\right.$$

From x−bω=0 and $y^{3}-\omega =0$, one obtains:

(4) $$x=by^{3},\;\omega =y^{3}$$

Substituting these expressions into c−xy−z=0 yields,

(5) $$z=c-by^{4}$$

Further substituting $\omega =y^{3}$ and $z=c-by^{4}$ into $a^{2}y+(c-by^{4})y^{3}=0$ gives:

(6) $$a^{2}y+(c-by^{4})y^{3}=0$$

(7) $$y(a^{2}+cy^{2}-by^{6})=0.$$

Let $u=y^{2}\left(u\geq 0\right)$. Then the equilibrium-point equation can be reduced to

(8) $$bu^{3}-cu-a^{2}=0.$$

Therefore, one equilibrium point is

(9) $$E_{0}=(0,0,c,0)$$

If $u_{*}$ denotes the positive real root of $bu^{3}-cu-a^{2}=0$, then the other two symmetric equilibrium points can be written as:

(10) $$E_{\pm }=\left(\pm bu_{*}^{3/2},\pm \sqrt{u_{*}},c-bu_{*}^{2},\pm u_{*}^{3/2}\right)$$

For the representative parameter set *a* = 1.6, *b* = 2.25, and *c* = 1.2, the equation:

(11) $$2.25u^{3}-1.2u-2.56=0$$

has the positive real root: $u_{*}\approx 1.21298467$,

Accordingly, the three equilibrium points are obtained as:

(12) $$\left\{\begin{array}{l} E_{0}=(0,0,1.2,0)\\ E_{+}\approx (3.005837,1.101356,-2.110497,1.335928)\\ E_{-}\approx (-3.005837,-1.101356,-2.110497,-1.335928) \end{array}\right..$$

The Jacobian matrix of the system is:

(13) $$J(x,y,z,\omega )=\left[\begin{array}{cccc} 0 & a^{2} & \omega & z\\ 1 & 0 & 0 & -b\\ -y & -x & -1 & 0\\ 0 & 3y^{2} & 0 & -1 \end{array}\right]$$

At the equilibrium point *E*_0_, the Jacobian matrix becomes:

(14) $$J(E_{0})=\left[\begin{array}{cccc} 0 & a^{2} & 0 & c\\ 1 & 0 & 0 & -b\\ 0 & 0 & -1 & 0\\ 0 & 0 & 0 & -1 \end{array}\right]$$

Its eigenvalues are: $\lambda_{1,2}=\pm a,\;\lambda_{3,4}=-1,-1$.

For *a* = 1.6, the eigenvalues are λ = {1.6, −1.6, −1, −1}. Hence, *E*_0_ is an unstable saddle point.

For the two nonzero equilibrium points *E*_+_ and *E*_−_, the eigenvalues are numerically obtained as:

(15) $$\lambda_{1,2}\approx 0.342116\pm 2.592439i,\;\lambda_{3,4}\approx -1.342116\pm 1.138666i.$$

Since both *E*_+_ and E_−_ have a pair of eigenvalues with positive real parts, these two equilibrium points are also unstable. Therefore, under the above parameter setting, all equilibrium points of the proposed system are unstable.

#### 2.1.2. Dissipativity Analysis

To characterize the dissipative nature of the system, the divergence of the associated vector field is evaluated as:

(16) $$\nabla \cdot f=\frac{\partial \dot{x}}{\partial x}+\frac{\partial \dot{y}}{\partial y}+\frac{\partial \dot{z}}{\partial z}+\frac{\partial \dot{\omega }}{\partial \omega }=\sum_{i}\frac{\partial {\dot{x}}_{i}}{\partial x_{i}}$$

For the proposed system, the divergence is a negative constant:

(17) ∇⋅f=0+0−1−1=−2<0

According to Liouville’s theorem, the evolution of an infinitesimal phase-space volume element *V*(0) satisfies:

(18) $$V(t)=V(0)\exp\left(\int_{0}^{t}\nabla \cdot fd\tau \right)=V(0)e^{-2t}$$

Hence, the phase-space volume contracts exponentially at a uniform rate, indicating that the system is strongly dissipative. This strong dissipativity implies that all trajectories are ultimately confined to a bounded region and are attracted to an attracting set, thereby providing a necessary foundation for the formation of strange attractors and other complex long-term dynamics.

The system contains multiple nonlinear terms and multiplicative coupling components, demonstrating strong nonlinear interaction among state variables. In particular, bilinear coupling terms (e.g., *-xy*) represent inter-variable feedback and energy exchange, while multiplicative coupling terms (e.g., *zw*) introduce parametric-like modulation that enhances stretching-and-folding behavior in phase space. Moreover, the cubic nonlinearity in $\overset{•}{\omega }=y^{3}-\omega$ makes *w* a first-order tracking (inertial) response to *y*^3^, and the resulting feedback of *w* into other state equations further reinforces closed-loop coupling and complex evolution. Collectively, these nonlinear coupling effects endow the system with rich bifurcation structures and the potential to exhibit chaotic dynamics.

### 2.2. Analysis of System Nonlinear Characteristics

#### 2.2.1. Two-Dimensional Phase Diagram

**Figure 1:**
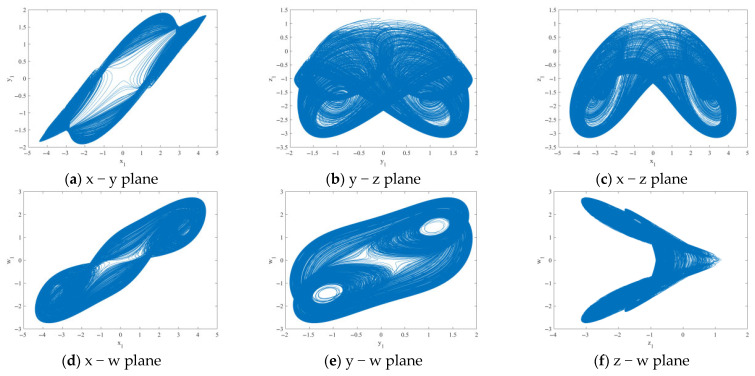
The two-dimensional phase diagram obtained when parameters *a* = 1.6, *b* = 2.25, and *c* = 1.2, and the initial conditions (*x*(0), *y*(0), *z*(0), *w*(0)) = (2, 0, 0, 0).

Phase portrait analysis is an effective means of directly observing the dynamical behavior of nonlinear systems. Therefore, researchers often construct phase portraits to visually determine system working status and dynamical characteristics, enabling rapid identification of system behavior without complex numerical calculations. This study is conducted under the conditions *a* = 1.6, *b* = 2.25, *c* = 1.2 with initial conditions (*x*(0), *y*(0), *z*(0), *w*(0)) = (2, 0, 0, 0). Figure 1 shows the two-dimensional phase diagram of the system.

#### 2.2.2. Lyapunov Exponential Spectrum and Kaplan–Yorke Dimension Analysis

1. Lyapunov exponential spectrum

**Figure 2:**
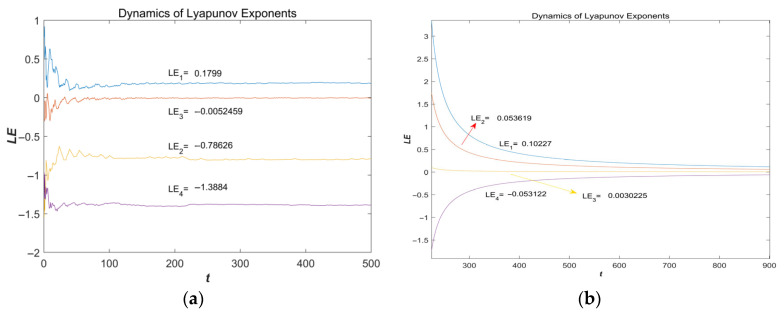
Lyapunov exponent spectrum (LEs) of the system. (**a**) The Lyapunov exponent spectrum (LEs) obtained with parameters *a* = 1.5, *b* = 2.25, and *c* = 1.2, and the initial conditions (*x*(0), *y*(0), *z*(0), *w*(0)) = (2, 0, 0, 0); (**b**) The Lyapunov exponent spectrum (LEs) obtained with parameters *a* = 0.5, *b* = 0.5, *c* = 2.5, and the initial conditions (*x*(0), *y*(0), *z*(0), *w*(0)) = (2, 0, 0, 0).

Figure 2a presents the Lyapunov exponent spectrum of the proposed system for *a* = 1.5, *b* = 2.25, *c* = 1.2 with initial conditions (*x*(0), *y*(0), *z*(0), *ω*(0)) = (2, 0, 0, 0). The obtained Lyapunov exponents are LE1 = 0.1799, LE2 = −0.78626, LE3 = −0.0052459, and LE4 = −1.3884. Since LE1 is positive, nearby trajectories diverge exponentially along at least one direction in the phase space, indicating sensitive dependence on initial conditions. Meanwhile, LE2 and LE4 are negative and LE3 is approximately zero, which is consistent with the behavior of continuous-time chaotic systems, where one exponent is typically close to zero. In addition, the sum of the Lyapunov exponents is negative, implying that the system is dissipative and that the overall motion is contracting in phase space, so trajectories remain bounded and evolve on an attractor—one of the key characteristics of dissipative chaotic systems.

Figure 2b shows the Lyapunov exponent spectrum for *a* = 0.5, *b* = 0.5, *c* = 2.5 with the same initial conditions (2, 0, 0, 0). The computed exponents are LE1 = 0.10227, LE2 = 0.052619, LE3 = 0.0030225, and LE4 = −0.053122. In this case, LE1 and LE2 are positive, LE3 remains very close to zero, and LE4 is negative. This indicates that under certain conditions, the system is capable of exhibiting hyperchaotic characteristics. This confirms that the system expands along at least two directions in phase space, leading to more complex dynamical behavior and a richer attractor structure.

2. Kaplan–Yorke Dimension Analysis

The Kaplan–Yorke dimension is a commonly used measure to estimate the fractal dimension of a chaotic attractor from its Lyapunov exponent spectrum. For a continuous-time dynamical system, after sorting the Lyapunov exponents in descending order as λ1 ≥ λ2 ≥ … ≥ λn, the Kaplan–Yorke dimension is defined as:

(19) $$D_{KY}=k+\frac{\sum_{i=1}^{k}\lambda_{i}}{\left|\lambda_{k+1}\right|}$$

where k is the largest integer satisfying $\sum_{i=1}^{k}\lambda_{i}\geq 0$. A fractional value of *D_KY_* indicates the fractal nature of the attractor, and a higher dimension implies richer dynamical complexity.

Based on the Lyapunov exponents presented, we computed the Kaplan–Yorke dimensions for two representative parameter sets.

For (*a* = 1.5, *b* = 2.25, *c* = 1.2), we obtained *D_KY_* = 2.2221; (*a* = 0.5, *b* = 0.5, *c* = 2.5), we obtained *D_KY_* = 4.

**Figure 3:**
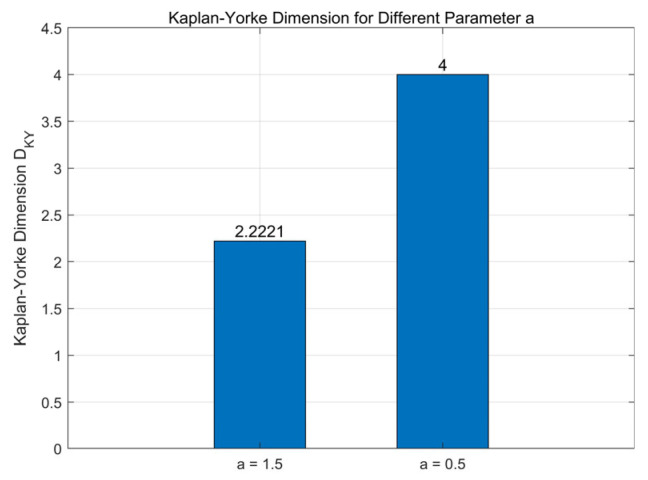
Kaplan–Yorke dimensions for Different Parameter *a* = 1.5 and *a* = 0.5.

Figure 3 presents a bar chart comparing the Kaplan–Yorke dimensions for these two cases. Both dimensions are fractional and exceed two, confirming that the attractor possesses a fractal structure and exhibits chaotic or hyperchaotic behavior. Notably, when *a* = 0.5, the Kaplan–Yorke dimension reaches a value of 4 in one of the cases, which is a key characteristic of conservative hyperchaotic systems, further confirming the hyperchaotic nature of the proposed system. These results further verify the rich dynamical behaviors of the proposed system and demonstrate its capability to generate highly complex chaotic sequences suitable for image encryption applications.

#### 2.2.3. Bifurcation Diagram

**Figure 4:**
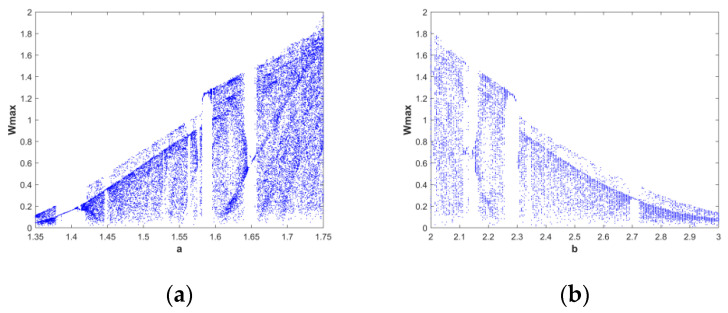
Bifurcation diagram system. (**a**) a − Wmax; (**b**) b − Wmax.

The bifurcation diagram a-wmax of the system with parameter *a* ∈ [1.35, 1.75], fixed parameters *b* = 2.25, *c* = 1.2 and initial conditions (*x*(0), *y*(0), *z*(0), *w*(0)) = (2, 0, 0, 0) is shown in Figure 4a. The bifurcation diagram shows a distinct chaotic—non-chaotic—chaotic state… At *a* ∈ [1.6, 1.64], *b* = 2.25, the system is in a chaotic state. The bifurcation diagram b-wmax obtained by the system with parameters *b* ∈ [2, 3], *a* = 1.6, *c* = 1.2, and the initial conditions (*x*(0), *y*(0), *z*(0), *w*(0)) = (2, 0, 0, 0) is shown in Figure 4b. As the value of b keeps increasing, the chaotic complexity of the system becomes lower and lower. It is a significant chaotic state when the system parameter *b* ∈ [2.2, 2.25] and *a* = 1.6.

#### 2.2.4. Poincare Cross-Sectional View

**Figure 5:**
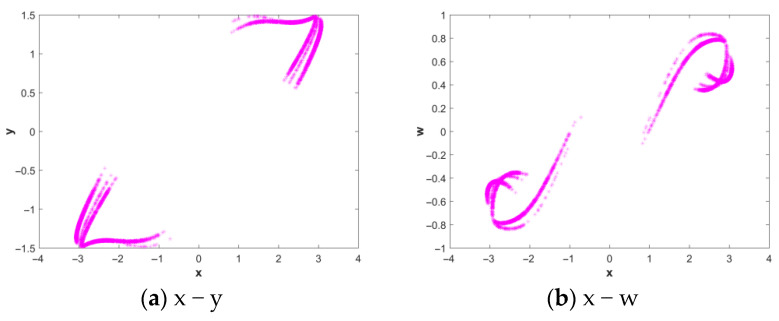
Poincare diagrams.

Figure 5 shows that the Poincare diagrams obtained for *a* = 1.6, *b* = 2.25, *c* = 1.2 and the initial conditions (*x*(0), *y*(0), *z*(0), *w*(0)) = (2, 0, 0, 0) are the Poincare diagrams of the proposed system (a) x − y, (b) x − w, where (a) is the Poincare graph of the x − y plane, and (b) is the Poincare graph of the x − w plane. The Poincare section shows a series of dense points with fractal structures in clusters, so the motion is chaotic.

#### 2.2.5. Chaotic Transient

With the above parameter setting, the system displays a typical transient-chaos phenomenon: it behaves chaotically for a short time and then rapidly converges to a periodic orbit, an equilibrium point, or another attractor. This “chaos first, then sudden change” evolution indicates an unstable transition in the system dynamics.

**Figure 6:**
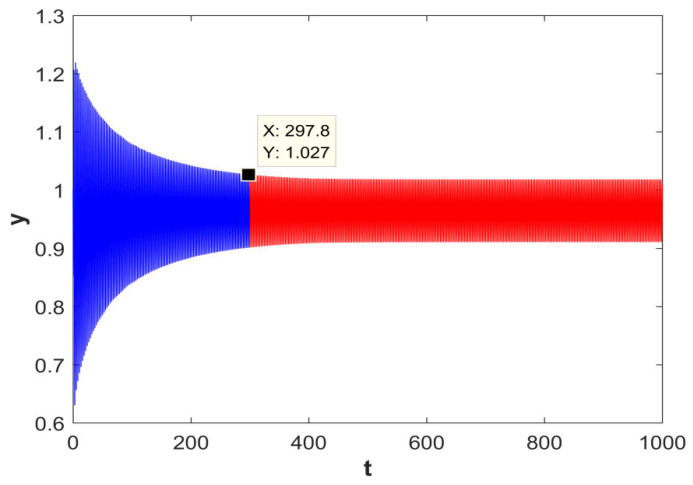
Transient state diagram.

Specifically, the parameters are set to (*a* = 1.2), (*b* = 2.7), and (*c* = 0.8), with initial conditions ((*x*(0), *y*(0), *z*(0), *w*(0)) = (2, 1, 0, 1)). The total simulation duration is 1000 s. As shown in Figure 6, the time series can be clearly divided into two stages, revealing an evident temporal difference between the chaotic regime and the subsequent periodic regime. According to the coordinates of the transition point marked in the figure, the system evolves in a chaotic state over (t ∈ (0, 297.8)), and then switches abruptly to a stable periodic state over (t ∈ (297.8, 1000)).

#### 2.2.6. Multiple Attractors

**Figure 7:**
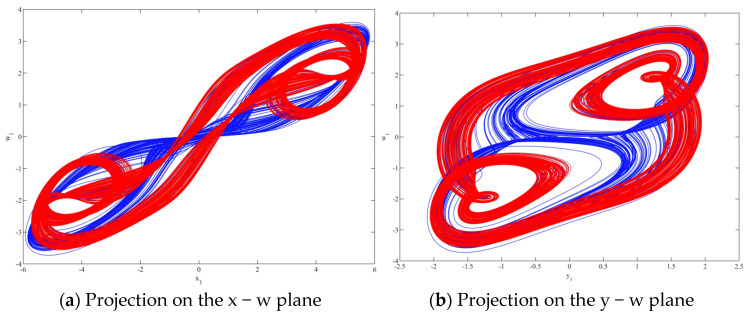
The projections of (2, 0, 0, 0) (blue) and (0, 0.17, 0, 0) (red) on the x − w and y − w planes under the condition of the same parameters but different initial values.

Based on the system parameters (*a* = 1.6, *b* = 2.25 and *c* = 3), Figure 7 illustrates the attractor projections on the x − w and y − w phase planes. In Figure 7, the blue trajectory (hidden attractor) is generated from the initial condition (2, 0, 0, 0), whereas the red trajectory is obtained from (0, 0.17, 0, 0). With changes in the system states and their corresponding parameter settings, the attractor trajectories exhibit pronounced transitions, indicating significant qualitative shifts in the system dynamics. Although the parameter variations are not symmetric, within a certain range, adjusting the parameters can produce attractor phase portraits with different scales while preserving similar trajectory patterns. In addition, when the first three components of the initial condition are fixed, varying only the fourth component for the red trajectory does not noticeably affect the resulting attractor, highlighting the system’s robustness and adaptability to perturbations in the initial state. Overall, these observations suggest that the system possesses multistability and is capable of generating multiple complex attractors that can coexist under the same parameter set.

#### 2.2.7. Spectral Entropy Analysis

**Figure 8:**
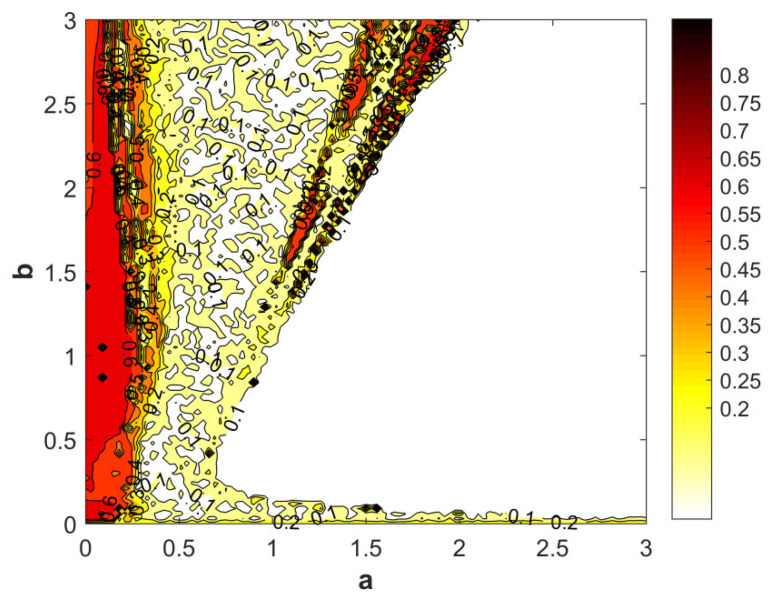
The chaotic characteristic graph based on spectral entropy complexity obtained with parameters *a* ∈ [0, 3], *b* ∈ [0, 3], *c* = 1.2 and the initial conditions (*x*(0), *y*(0), *z*(0), *w*(0)) = (2, 0, 0, 0).

Spectral entropy (SE) plots are widely used in signal processing and nonlinear dynamics as a visual measure of complexity, capturing variations in a system’s chaotic intensity and information entropy across different parameter settings or time scales. With parameters fixed at (*a* ∈ [0, 3]) and (*b* ∈ [0, 3]), and (*c* = 1.2), the corresponding spectral entropy contour map is shown in Figure 8. In the figure, the dark-red regions exhibit SE values of approximately 0.8 and are broadly scattered, indicating a pronounced chaotic regime. The red regions correspond to SE values around 0.6 and display a more concentrated distribution, suggesting quasi-periodic behavior. The yellow regions, with SE values near 0.2, are highly continuous and concentrated, reflecting a periodic state. Overall, the spectral entropy varies with the parameter, implying that as the parameter increases, the system gradually loses entropy and becomes more regular: chaotic behavior weakens while stability is enhanced.

### 2.3. Circuit Design and Implementation

We will define a time variable τ, let $\tau =\tau_{0}t$, $\tau_{0}=1/RC$. The new equation is obtained as follows:

(20) $$\left\{\begin{array}{l} \frac{dx}{d\tau }=\frac{1}{RC}(a^{2}y-zw)\\ \frac{dy}{d\tau }=\frac{1}{RC}(x-bw)\\ \frac{dz}{d\tau }=\frac{1}{RC}(c-xy-z)\\ \frac{dw}{d\tau }=\frac{1}{RC}(y^{3}-w) \end{array}\right.$$

**Figure 9:**
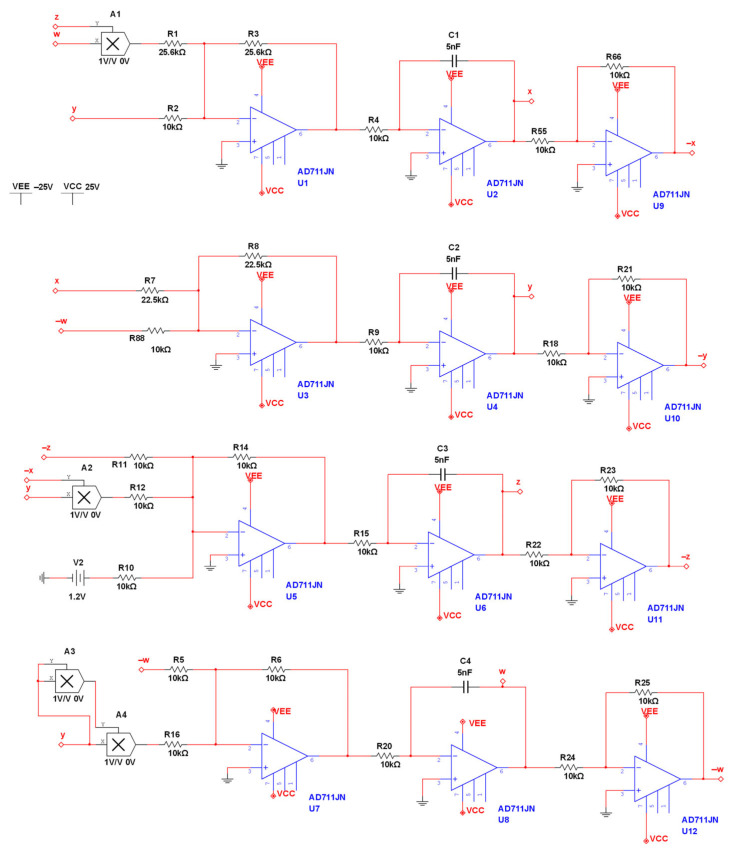
Schematic diagram of the chaotic circuit.

Based on the mathematical model of the system formula and in accordance with Kirchhoff’s law, we obtained the corresponding circuit schematic diagram as shown in Figure 9. In the schematic diagram, we used 12 AD711JN operational amplifiers, 4 MULTIPLIER multipliers, 25 linear resistors, 4 capacitors and a power supply (±25 V) (Analog Devices, Wilmington, MA, USA). For *a* = 1.6, *b* = 2.25, *c* = 1.2, according to circuit theory knowledge, the values of each component in the circuit are:

R_i = 10 kΩ, 4, 55, 66, 88, 9, 18, 21, 11, 10, 12, 14, 15, 22, 23, 5, 16, 6, 20, 24, 25(I = 2);

R_i = 25.6 kΩ(i = 1, 3, 7, 8), C_i = 5nF(i = 1, 2, 3, 4).

**Figure 10:**
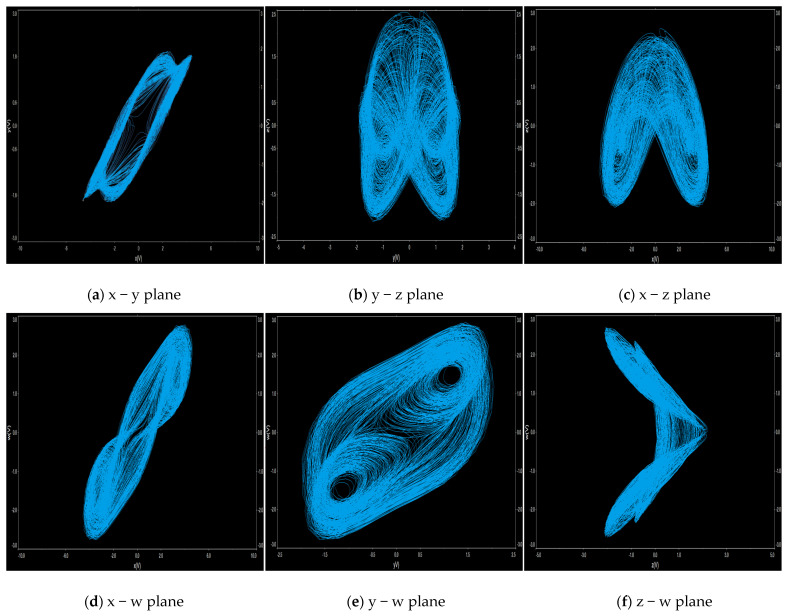
Simulation phase diagram of the analog circuit.

We use the software of Multisim 14.0 to build circuits. The experimental results are shown in Figure 10a–f. When *a* = 1.6, it is consistent with the numerical simulation results shown in the phase diagram.

## 3. Based on Hyperchaotic Systems and DNA-Encoded Telemetry Graph Encryption Algorithms

**Figure 11:**
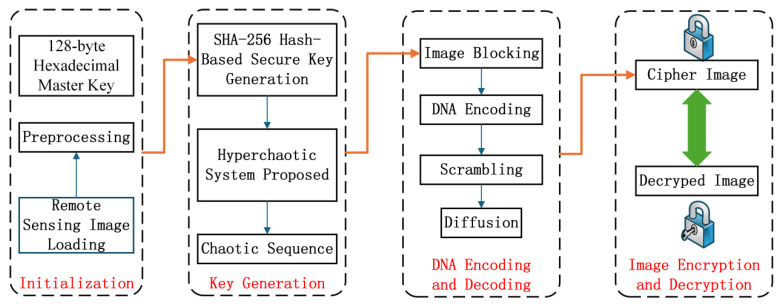
Flowchart of the encryption algorithm.

By integrating the proposed four-dimensional hyperchaotic system with DNA encoding and Secure Hash Algorithm 256 (SHA-256) hash technology, a highly secure encryption and decryption scheme tailored for remote sensing images is realized. The algorithm initiates with the preprocessing of a 128-byte hexadecimal master key, from which a cryptographically robust key is derived via the SHA-256 hash function; this key subsequently drives the hyperchaotic system to generate high-entropy chaotic sequences for cryptographic operations. The encryption pipeline encompasses image blocking, DNA encoding, scrambling (position permutation), and pixel diffusion, synergistically enhancing confusion and diffusion properties to resist cryptanalytic attacks. The decryption process reverses these procedures—including DNA decoding, inverse scrambling, and inverse diffusion—to ensure lossless recovery of the original remote sensing image. Figure 11 depicts the algorithm flowchart, and detailed elaboration of each phase is provided in subsequent sections.

### 3.1. Data Initialization and Image Preprocessing

In the key and image loading stage, a 128-bit hexadecimal key is first defined. This key serves as the core credential for encryption and decryption processes, providing the initial security parameter for the entire system. Subsequently, the original RGB image is loaded through the image reading function. Unlike traditional two-dimensional images, RGB images are multi-channel. Functions are employed to determine the numbers of rows (M), columns (N), and channels, thereby clarifying the basic dimensional information. Generally, the image comprises three channels: R, G, and B. To meet the processing requirements of subsequent encryption algorithms, the original image is converted into double-precision format, and data from each channel are extracted as the plaintext for encryption, laying the foundation for subsequent operations.

### 3.2. Key Generation

#### 3.2.1. Hash Algorithm

In the key processing stage, the system binds image features with the key through multi-level hash operations, generating a 256-bit hash value with strong randomness [26]. The final key is produced through base conversion and XOR operations. The specific steps are as follows:

Step 1: Calculate the feature values of the image

Perform summation operations on the rows, columns, and diagonals of the image. Sum the image matrix by rows to obtain the column vector *SumRow* and sum the image matrix by columns to obtain the row vector *SumCol*, which is then transposed to a column vector. Use spdiags and rot90 to extract the diagonal elements of the image and sum them. The formula is:

(21) $$\left\{\begin{array}{c} SumRow={\left[\sum_{j=1}^{N}\mathrm{PlainImg}(i,j)\right]}_{M\times 1},\;i\in [1,M]\\ SumCol={\left[\sum_{i=1}^{M}\mathrm{PlainImg}(i,j)\right]}_{1\times N}^{\prime },\;j\in [1,N]\\ SumDiag=\sum (\mathrm{spdiags}(\mathrm{rot}90(\mathrm{PlainImg}))) \end{array}\right.$$

Among them, PlainImg(*i*, *j*) represents the pixel values of the *i*-th row and *j*-th column of the image, rot90 indicates a 90° rotation of the image, and spdiags indicates the extraction of diagonal elements.

Step 2: Multi-level hash mixing

The first level first calculates the Message-Digest Algorithm 5 (MD5) hash for the row sum, column sum, and diagonal sum (*SumDiag*), respectively. The second level concatenates three MD5 hash values and calculates the SHA-256 hash to obtain the hash value of a 256-bit binary number.

Step 3: Base system conversion

Convert the 128-bit hexadecimal master key to a binary sequence and convert every 8 bits of binary to a decimal number to obtain the decimal array *HEX_D_* (with 16 elements). Then perform the same transformation on the 256-bit hash value obtained in Step 2 to obtain the decimal array *Hash_D_* (with 32 elements). To solve the inconsistency in the length of the two arrays, we extend *HEX_D_* to 32 elements by repeated concatenation to align its dimension with *Hash_D_* before performing subsequent operations.

Dimension alignment preprocessing: Since the number of elements in *HEX_D_* (16 elements) is inconsistent with that in *Hash_D_* (32 elements), we extend *HEX_D_* to 32 elements by repeating and concatenating it twice, so that its dimension is consistent with *Hash_D_*. After the dimension alignment, the XOR operation can be performed normally.

Step 4: XOR mixing generates the final key *Key_D_*

Perform the XOR operation on the two decimal arrays in Step 3, and the formula is as follows:

(22) $$Key_{D}=HEX_{D}⊕Hash_{D}$$

Step 5: Extract the key feature *Key_F_*

The global eigenvalues are calculated through bitwise XOR, and the formula is as follows:

(23) $$Key_{F}=Key_{D}\left(1\right)⊕Key_{D}\left(2\right)⊕\ldots ⊕Key_{D}\left(n\right)$$

In Equation the variable *n* represents the total number of segmented keys *Key_D_* involved in the bitwise XOR operation. Its value is jointly determined by the algorithm design and the input data dimension: in the proposed scheme of this paper, *n* is equal to the number of key segments generated by the iteration of the chaotic system, or equivalently the dimensional length of the hash output result. It indicates that all *n* segmented keys are sequentially subjected to bitwise XOR operation and finally merged to obtain the global feature key *Key_F_*.

#### 3.2.2. Generation of Chaotic Sequences

The four-dimensional hyperchaotic system proposed in this paper has undergone extensive analysis and verification in preceding sections. It is a high-dimensional chaotic system exhibiting complex dynamical behavior, extreme sensitivity to initial conditions (butterfly effect), and generating sequences with favorable pseudo-randomness. Consequently, this system is employed to generate pseudo-random sequences. The differential equations of Equation (2) are described as:

(24) $$\left\{\begin{array}{l} \frac{dx}{dt}=a^{2}y+z\omega \\ \frac{dy}{dt}=x-b\omega \\ \frac{dz}{dt}=c-xy-z\\ \frac{d\omega }{dt}=y^{3}-\omega \end{array}\right.$$

Among them, *a* = 1.6, *b* = 2.25, and *c* = 1.2.

The next step is to discretize the iterative formula, which can be achieved through Euler discretization:

(25) $$\left\{\begin{array}{l} x(i)=\alpha^{2}y(i-1)+z(i-1)\omega (i-1)\\ y(i)=x(i-1)-b\omega (i-1)\\ z(i)=c-x(i-1)y(i-1)-z(i-1)\\ \omega (i)=y{\left(i-1\right)}^{3}-\omega (i-1) \end{array}\right.$$

The global key feature value *KeyF* is mixed and calculated with the decimal key segment data *d_k_* extracted from the master key to generate the initial value. The mixing formula is as follows:

(26) $$\left\{\begin{array}{l} x(1)=\frac{{⊕}_{k=1}^{8}d_{k}⊕Key_{F}}{256}\\ y(1)=\frac{{⊕}_{k=9}^{16}d_{k}⊕Key_{F}}{256}\\ z(1)=\frac{{⊕}_{k=17}^{24}d_{k}⊕Key_{F}}{256}\\ \omega (1)=\frac{{⊕}_{k=25}^{32}d_{k}⊕Key_{F}}{256} \end{array}\right.$$

During chaotic sequence generation, state variable values produced by system iteration may be unevenly distributed. Normalizing the value range maps the chaotic sequence to the interval [0, 1), while an amplification factor prevents decimal precision loss and ensures uniformity and randomness in subsequent quantization. The formula is as follows:

(27) $$\left\{\begin{array}{c} x_{\mathrm{norm}}(i)=(x(i)\times 10^{4})-\left\lfloor x(i)\times 10^{4}\right\rfloor \\ y_{\mathrm{norm}}(i)=(y(i)\times 10^{4})-\left\lfloor y(i)\times 10^{4}\right\rfloor \\ z_{\mathrm{norm}}(i)=(z(i)\times 10^{4})-\left\lfloor z(i)\times 10^{4}\right\rfloor \\ \omega_{\mathrm{norm}}(i)=(\omega (i)\times 10^{4})-\left\lfloor \omega (i)\times 10^{4}\right\rfloor \end{array}\right.$$

Here, $\left\lfloor \cdot \right\rfloor$ it indicates rounding down.

The normalized value is used to quantify the generated key stream key:

(28) $$key=\;\mathrm{mod}\;\left(\left\lfloor 4\times \left[\begin{array}{c} x_{\mathrm{norm}}(i)\\ y_{\mathrm{norm}}(i)\\ z_{\mathrm{norm}}(i)\\ \omega_{\mathrm{norm}}(i) \end{array}\right]\right\rfloor ,4\right)$$

Among them, *key* ∈ {0, 1, 2, 3}, corresponding to the DNA bases encoding A, C, G, and T.

### 3.3. DNA Encoding and Decoding Algorithm

**Figure 12:**
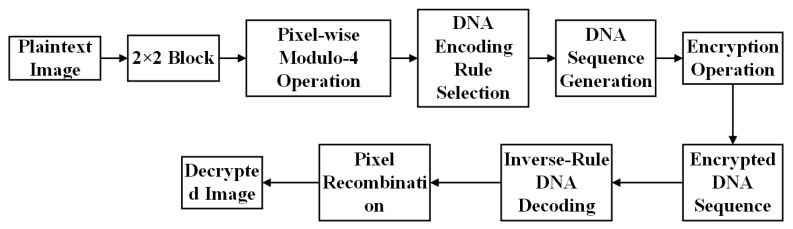
Flowchart of the DNA Encoding and Decoding Algorithm.

DNA-based image encryption is a technique that first maps pixel values of digital images into DNA base sequences (A, C, G, T), then employs DNA operation rules and chaotic systems to perform scrambling and diffusion and finally decodes sequences back to pixel values. This process comprises four key stages: block processing, dynamic rule selection, base mapping, and diffusion. The algorithm flowchart is shown in Figure 12.

#### 3.3.1. Base Coding Rules

The core of DNA encoding and decoding is to map binary data to a combination of four bases. Since each base can represent two-bit binary information (00, 01, 10, 11), there are multiple possible mapping relationships. Theoretically, there are a total of 4! = 24 possible combinations of the four bases, but in practical applications, 8 standard coding rules are usually adopted, as shown in Table 1.

#### 3.3.2. Encoding Process

DNA encoding is the process of converting plaintext data slices into DNA sequences, that is, encrypting the image and dividing the M × N image matrix into 2 × 2 sub-blocks:

Among them, *p_x_*,*_y_* represent the pixel values of the original image at the coordinates (*x*,*y*). For a 512 × 512 image, 65,536 DNA-encoded blocks will be generated.

(29) $$B_{i,j}=\left[\begin{array}{cc} p_{2i,2j} & p_{2i,2j+1}\\ p_{2i+1,2j} & p_{2i+1,2j+1} \end{array}\right],\;\left\{\begin{array}{l} 0\leq i<\frac{M}{2}\\ 0\leq j<\frac{N}{2} \end{array}\right.$$

According to Table 1, the system has predefined 8 DNA coding rules, and the rule selection formula is provided:

(30) $$RuleID=\mathrm{mod}\left(Key_{F},8\right)+1$$

According to the rule formula, encode each pixel value *p*:

(31) $$DNA(p)=\left\{\begin{array}{ll} A, & \mathrm{if}\;\mathrm{mod}(p,4)=0\\ T, & \mathrm{if}\;\mathrm{mod}(p,4)=1\\ C, & \mathrm{if}\;\mathrm{mod}(p,4)=2\\ G, & \mathrm{if}\;\mathrm{mod}(p,4)=3 \end{array}\right.$$

#### 3.3.3. Decoding Process

This process can be understood as the reverse process of DNA encoding, reversing the DNA sequence back to pixel values. First, convert the DNA sequence into a numerical map and then reverse map it according to the rules used during encryption to obtain the following formula:

(32) $$p=\left\{\begin{array}{ll} 0, & \mathrm{if}\;DNA=A\\ 1, & \mathrm{if}\;DNA=T\\ 2, & \mathrm{if}\;DNA=C\\ 3, & \mathrm{if}\;DNA=G \end{array}\right.$$

Then convert the obtained quaternary sequence into decimal pixel values and reorganize the data image through rules.

## 4. Performance Analysis of Image Encryption Algorithms

**Figure 13:**
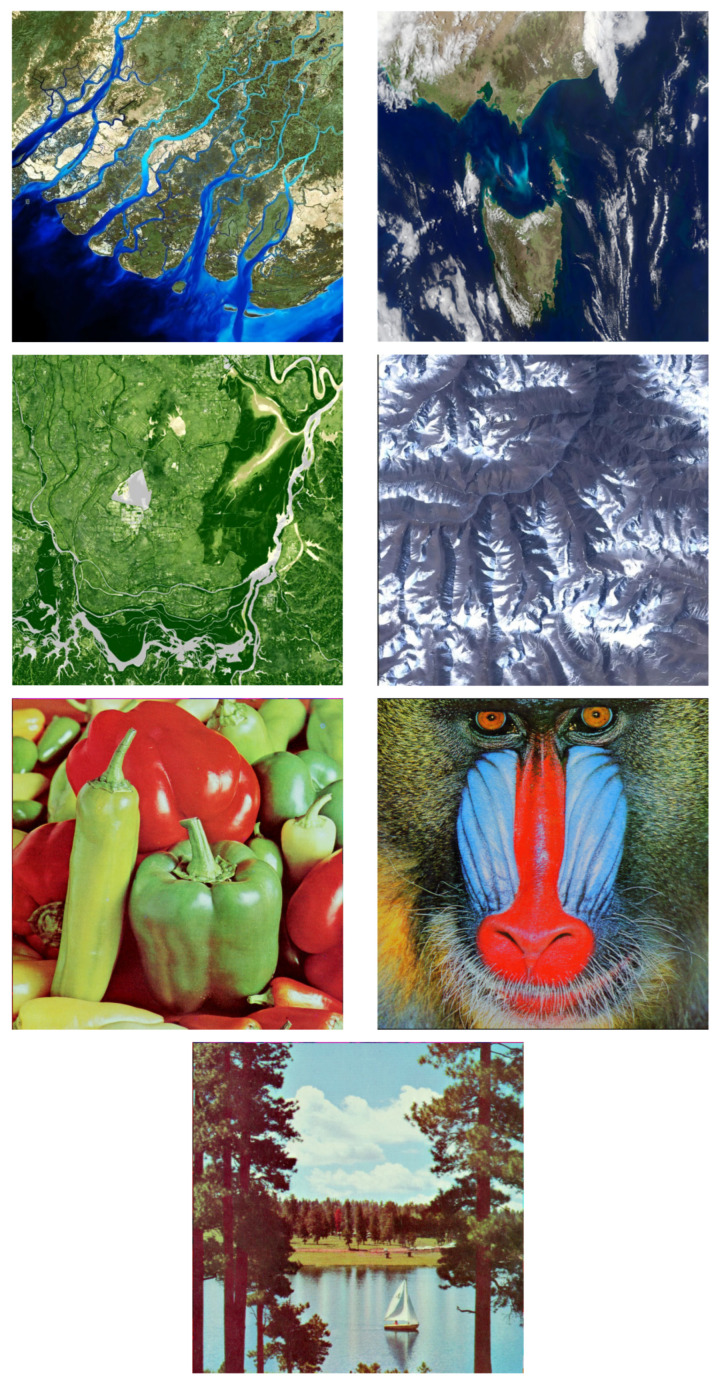
Original images Sea, Meteorology, Vegetation, Land, Pepper, Baboon, and Sailboat (512 × 512).

To verify the effectiveness and performance of the above-mentioned algorithm, this paper will conduct an analysis from several aspects such as histogram, information entropy, correlation, NPCR, UACI, and PSNR. Firstly, the images are encrypted through the above algorithm. The resolutions of the original images, including Sea, Meteorology, Vegetation, Land, Pepper, Baboon, and Sailboat, are all 512 × 512. The image data sources are all publicly available data on the Internet. The unprocessed original images are shown in Figure 13:

### 4.1. Histogram Analysis

**Figure 14:**
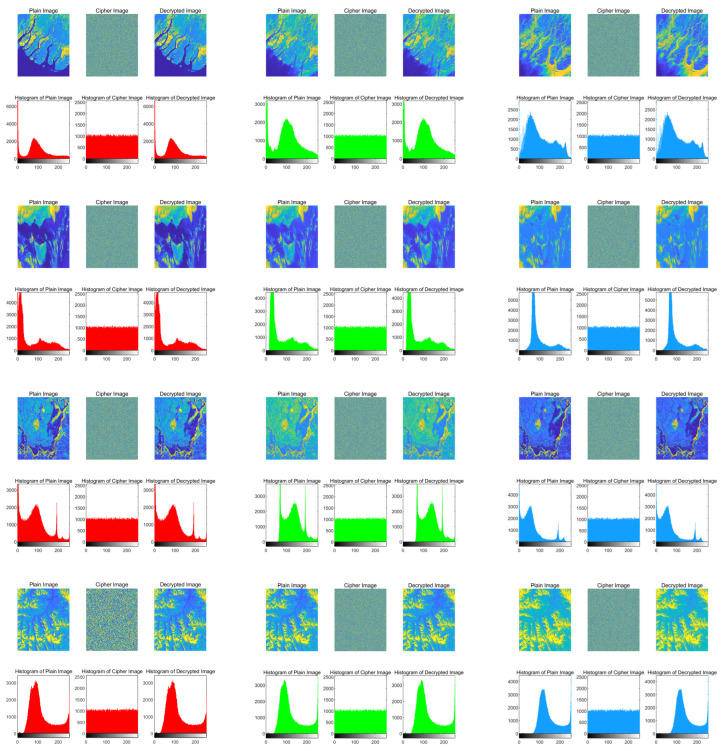

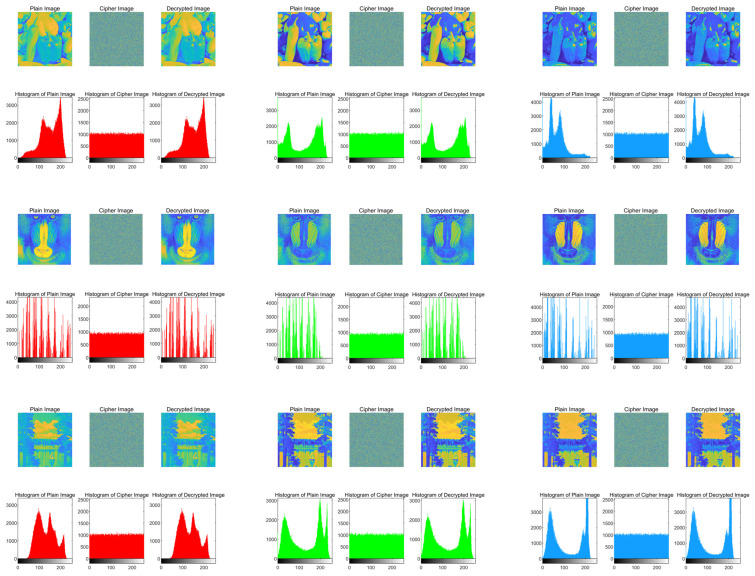
A histogram that separates the RGB three channels of Sea, Meteorology, Vegetation, and Land images. The red histogram represents the R channel in RGB; the green histogram represents the G channel in RGB; the blue histogram represents the B channel in RGB.

A histogram is a statistical tool that reflects data distribution patterns, visually displaying the distribution of quality characteristics and facilitating overall analysis. Generally, histograms of encrypted images should be uniformly distributed, whereas those of original images exhibit non-uniform distributions. Therefore, this study performs histogram calculations on original, encrypted, and decrypted images of the following RGB image groups. First, each image is separated into three channels: R, G, and B, as shown in Figure 14. The figure demonstrates that pixel value distributions of encrypted images are relatively uniform, while histogram distributions of decrypted images are highly similar to those of original images. This indicates that the proposed algorithm resists statistical attacks and restores images effectively.

### 4.2. Correlation Analysis

There is a high degree of correlation among pixels in plaintext images. Through encryption algorithms, this correlation can be reduced. Ideally, the encryption correlation should be 0, that is, the closer the correlation is to 0, the better the effect. Suppose $N\left(i=1,2,\ldots ,N\right)$ pairs of adjacent pixels are randomly selected in the image. The calculation formula for the correlation coefficient between the gray value $u=\left\{u_{i}\right\}$ and $v=\left\{v_{i}\right\}$ is as follows:

(33) $$\left\{\begin{array}{l} r_{xy}=\frac{\mathrm{cov}\left(u,v\right)}{\sqrt{D\left(u\right)}\sqrt{D\left(v\right)}}\\ \mathrm{cov}\left(u,v\right)=\frac{1}{N}\sum_{i=1}^{N}\left(x_{i}-E\left(u\right)\right)\left(y_{i}-E\left(v\right)\right)\\ D\left(u\right)=\frac{1}{N}\sum_{i=1}^{N}{\left(u_{i}-E\left(u\right)\right)}^{2}\\ E\left(u\right)=\frac{1}{N}\sum_{i=1}^{N}\left(u_{i}\right) \end{array}\right.$$

**Figure 15:**
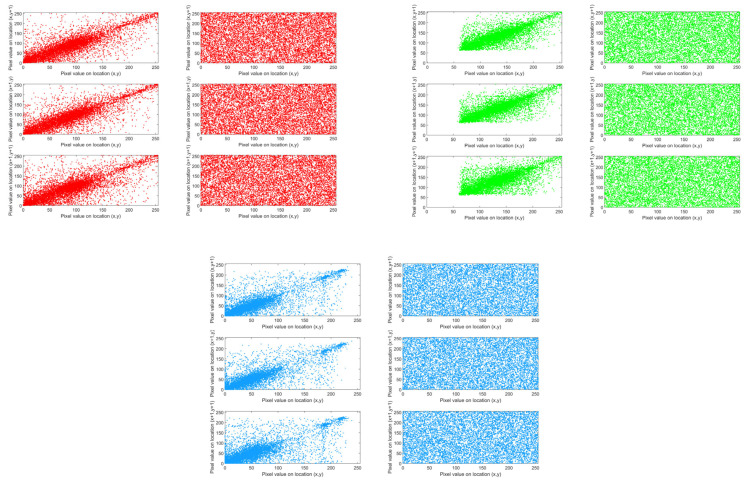
The correlation graph of the RGB channels in the Vegetation image.The red correlation graph represents the R channel in RGB; the green correlation graph represents the G channel in RGB; the blue correlation graph represents the B channel in RGB.

Based on the above formula, the images before and after encryption were calculated and analyzed. Taking the Vegetation image as an example, the correlations of these pixels in three directions (horizontal, vertical and diagonal) were studied. Figure 15 clearly shows the changes in the correlation of adjacent pixels before and after image encryption in the R, G, and B channels in these three directions. The analysis results clearly indicate that the unencrypted original image shows strong pixel correlation in the horizontal, vertical, and diagonal directions. After encryption, the correlations in all three directions decreased significantly.

Table 2 provides a detailed comparison of correlation coefficients for the images (Ocean, Climate, Vegetation, Land, Pepper, Baboon, and Sailboat) before and after encryption across three dimensions: horizontal, vertical, and diagonal. The data demonstrate that correlation coefficients in all directions approach 1 prior to encryption, indicating highly regular and predictable pixel distributions. However, after encryption using the proposed chaotic system, these coefficients are significantly reduced, approaching 0. This indicates that correlations in all directions have been effectively disrupted, and pixel distributions tend toward randomness, resisting statistical inference. The significant reduction in correlation coefficients demonstrates that the chaotic system effectively disrupts pixel correlations and enhances image randomness, thereby preventing information leakage.

### 4.3. Information Entropy Analysis

Image information entropy is a key indicator for quantifying the richness of image information. It is based on the concept of entropy in information theory and is used to evaluate the randomness and uncertainty of the distribution of image pixel values. This indicator reflects the complexity of the information contained in the image by analyzing the statistical characteristics of the grayscale or color values of the image: the higher the entropy value, the more uniform the distribution of pixel values, and the richer the information contained in the image. The lower the entropy value, the more concentrated the displayed pixel values tend to be, and the higher the information redundancy. Its calculation process consists of four core steps: (1) Establishing a probability distribution model of pixel values; (2) Calculate the amount of information at each gray level; (3) Obtain the expected value of the amount of information; (4) The final calculation is carried out through the Shannon entropy formula, where *P*(*x_i_*) represents the occurrence probability of each gray level. This indicator has significant application value in fields such as image encryption, compression and quality assessment.

(34) $$H(X)=-\sum_{i=1}^{n}p(x_{i})\log_{2}p(x_{i})$$

As shown in Table 3, comparison of information entropy before and after encryption reveals a clear trend: encrypted images exhibit significantly higher entropy than original images. This indicates that the encryption process effectively enhances uniformity in pixel value distribution, thereby increasing information capacity. Increased entropy is an important indicator of encryption quality, as it reflects uncertainty and randomness, playing a crucial role in preventing information leakage and enhancing security. The entropy values achieved by the proposed algorithm are significantly superior to those of existing schemes reported in the literature. Consequently, attackers can hardly capture useful information from statistical features, providing stronger security protection for image data.

### 4.4. Analysis of Differential Attack

Differential attack is an important technique in cryptanalysis. Its core principle involves compromising the encryption mechanism by observing response characteristics of the encryption system to subtle input data modifications. In image encryption, this attack manifests as the attacker deliberately modifying local pixels (typically single or a few pixels) of the original image and inferring statistical characteristics of the encryption algorithm by comparing differences between images before and after encryption.

To quantitatively evaluate encryption algorithm resistance to differential attacks, researchers have proposed two key indicators:

(1) Pixel change Rate (NPCR): This indicator precisely calculates the proportion of changes in the corresponding pixel values of the image before and after encryption. The higher the NPCR value, the stronger the sensitivity of the encryption algorithm to input changes, and the better the algorithm’s ability to resist differential attacks.
(2) Uniform Attack Coefficient Index (UACI): As a supplementary indicator to NPCR, UACI not only examines the proportion of pixel value changes but also comprehensively considers the amplitude of pixel value changes and the degree of alteration in the overall image structure, thereby providing a more comprehensive security assessment.

The ideal values of NPCR and UACI are 0.9961 and 0.3346, respectively, and the specific calculation formulas are as follows:

(35) $$\mathrm{NPCR}=\frac{1}{M\times N}\sum_{i=1}^{M}\sum_{j=1}^{N}D(i,j)\times 100\%$$

(36) $$\mathrm{UACI}=\frac{1}{M\times N}\sum_{i=1}^{M}\sum_{j=1}^{N}\frac{\left|C_{1}(i,j)-C_{2}(i,j)\right|}{255}\times 100\%$$

Table 4 compares the NPCR and UACI values of different images (Sea, Meteorology, Vegetation, Land, Pepper, Baboon, and Sailboat) under 1-bit and 2-bit differential attacks. The results show that the NPCR values of all images in the RGB three channels are close to the ideal value of 0.9961, indicating that the encryption algorithm is highly sensitive to input changes and has strong diffusivity. The UACI value is stable at around 0.334, close to the theoretical optimal value of 0.3346, indicating that the algorithm can evenly distribute the intensity of pixel variation and effectively resist differential attacks. It is worth noting that the NPCR and UACI modified by 2-bit are comparable to those of 1-bit, and in some cases, the values are even better, further verifying the robustness of the algorithm against larger disturbances. Overall, this encryption scheme demonstrates excellent resistance to differential attacks in both attack modes, meeting strict security requirements.

Table 5 shows the NPCR and UACI values of other encryption algorithms. Compared with the algorithm proposed in this paper, both NPCR and UACI have their own advantages and disadvantages. However, the algorithm proposed in this paper is relatively close to the ideal values under both parameters, with balanced performance.

### 4.5. Analysis of Clipping Attack and Salt-and-Pepper Noise Attack

Cropping attack is a structured occlusion attack. Its essence involves artificially inducing local integrity damage to image data by applying spatial domain masking operations to the ciphertext. Specifically, attackers force pixel values in specific ciphertext regions to zero according to a preset cropping ratio, thereby simulating data packet loss or malicious tampering during transmission or storage. The core of this attack lies in testing the redundant protection capability of the encryption algorithm for image structural information—that is, whether the decryption process can restore recognizable original content by exploiting the algorithm’s diffusion mechanism and key sensitivity when prominent ciphertext regions are removed.

Salt-and-pepper noise attack is a random impulse interference attack. Its mechanism involves injecting sparsely distributed extreme impulse noise into the ciphertext to simulate sudden channel errors or sensor failures. Mathematically, this attack is represented as a random process driven by a probability density function, where the noise density parameter directly determines the spatial distribution of contaminated pixels.

**Figure 16:**
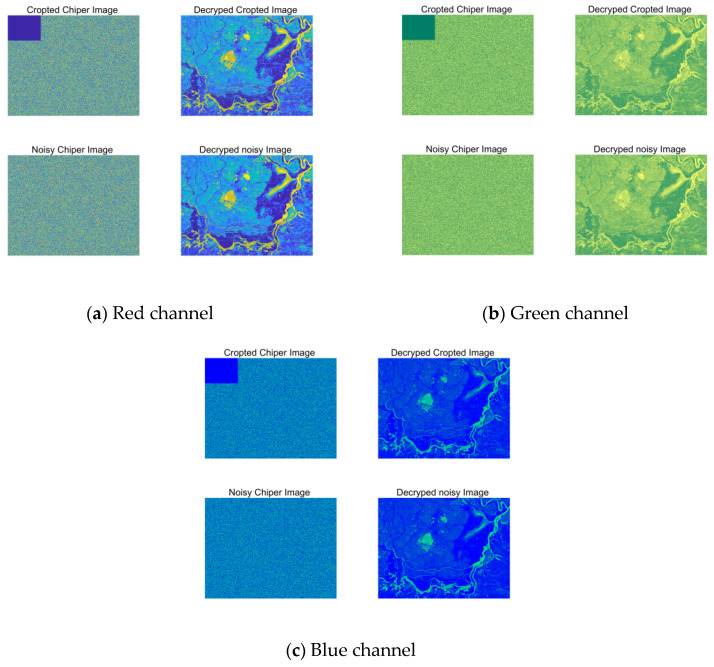
Analysis of cropping attacks and noise attacks under the RGB three channels of the Vegetation image.

The peak signal-to-noise ratio (PSNR) is an objective metric for measuring image or signal quality. It assesses quality loss by comparing differences between the original and reconstructed images. PSNR serves here as a quantitative indicator of anti-noise performance: higher values indicate stronger ability to suppress random impulse interference and higher decrypted image fidelity. This study conducts simulation experiments using a cropping attack with ratio 1/16 and salt-and-pepper noise attack with density parameter 0.005. The results are shown in Figure 16, demonstrating that the proposed encryption algorithm effectively resists cropping and salt-and-pepper noise attacks, with more significant effectiveness observed under salt-and-pepper noise conditions.

To better verify the performance of the algorithm, the PSNR value will be calculated through a formula for comparison. The specific formula is as follows:

(37) $$MSE=\frac{1}{m\times n}\sum_{i=1}^{m}\sum_{j=1}^{n}(P(i,j)-D(i,j){)}^{2}$$

(38) $$PSNR=10\times \log_{10}\left(\frac{255^{2}}{MSE}\right)$$

The above formula is implemented through the mean square error (MSE), where *P*(*i*, *j*) represents the pixel values of the original image and *D*(*i*, *j*) represents the pixel values of the reconstructed image. MSE reflects the overall difference between two images. The smaller the value, the higher the similarity. The calculation results are shown in Table 6. It can be seen that under salt-and-pepper noise attack, the PSNR value of the image can be guaranteed to be above 30 db, indicating that the image can resist salt-and-pepper noise attack well. Under the clipping attack, although the PSNR drops, it basically remains around 20 db, and the algorithm can resist the clipping attack to a certain extent.

### 4.6. Decryption Process and Key Handling

In the proposed encryption scheme, the generation of the key stream depends on the hash code of the plain image, which is computed using SHA-256 via the HashSumRowSumCol function. This hash code is combined with the user-provided key (KeyHex) to produce the final KeyImage used for encryption (see Section 3). However, during decryption, the receiver does not have access to the original plain image and therefore cannot recompute the same hash code. To address this issue, we adopt a practical key handling strategy: the key stream itself, together with the auxiliary parameters, is saved during encryption and securely transmitted to the receiver. Specifically, after encryption, we store the following variables in a .mat file.

At the receiver side, these variables are loaded from the .mat file and directly passed to the Decryption function. The decryption algorithm then applies the inverse operations to recover the plain image from the ciphertext, without ever needing the original hash. This approach ensures correct decryption while maintaining security, because the key stream itself is derived from the plaintext hash and the secret key, and it is assumed to be transmitted over a secure channel.

**Figure 17:**
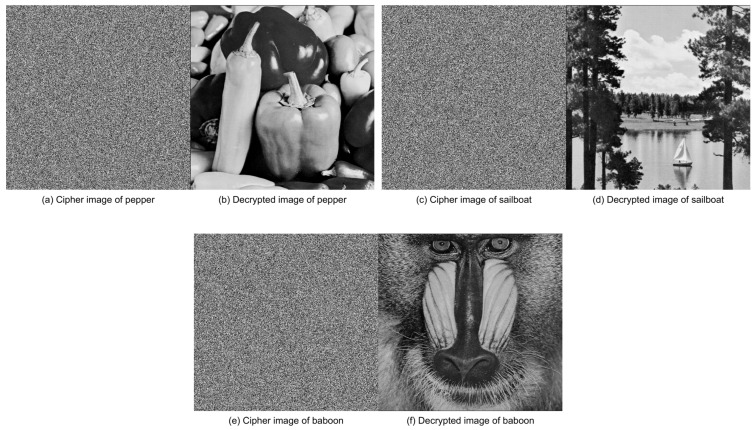
Encryption and decryption results for three test images. (**a**) Cipher image of pepper; (**b**) Decrypted image of pepper; (**c**) Cipher image of sailboat; (**d**) Decrypted image of sailboat; (**e**) Cipher image of baboon; (**f**) Decrypted image of baboon. Only the green channel of each color image is processed.

We have validated this procedure on three standard test images: pepper, sailboat, and baboon. For simplicity, only the green channel (G) of each color image is processed; the other two channels can be treated analogously. Figure 17 shows the cipher and decryption results for these three images. As can be observed, each decrypted image is visually identical to its original plain version, confirming the correctness of the decryption process. These experimental results demonstrate that our key handling method successfully recovers the plain image at the receiver side without requiring the original hash.

## 5. Conclusions

This paper presents a strongly dissipative, nonlinearly coupled four-dimensional hyperchaotic system and applies it to RGB image encryption. The dynamical properties of the proposed system are systematically investigated through fixed-point analysis, dissipativity analysis, phase portraits, Lyapunov exponents, Kaplan–Yorke dimension analysis, bifurcation analysis, Poincare sections, chaotic transients, attractors, and spectral entropy. Simulation results verify that the system exhibits hyperchaotic behavior within a specified parameter range and displays complex nonlinear dynamic characteristics. Notably, spectral entropy analysis quantifies the high randomness of the system, establishing a direct correlation between its dynamic complexity and cryptographic security. The uniform spectral distribution of the system contributes to evenly distributed ciphertext histograms and near-ideal information entropy in the encrypted results, thereby strengthening resistance to spectral analysis and pattern-based attacks. To verify practical implementability, an analog circuit is designed in Multisim 14.0, and circuit-level simulations further confirm the feasibility and effectiveness of the proposed system. Beyond hardware validation, this circuit implementation demonstrates robust performance under finite-precision conditions and component tolerances, confirming that the system maintains hyperchaotic behavior in physical realizations and exhibits potential for high-speed hardware-based encryption—critical for large-scale remote sensing image processing. Four chaotic sequences generated by the system under given initial conditions, combined with hash values and DNA encoding rules, are utilized for RGB image encryption. Performance evaluations based on histogram distribution, information entropy, adjacent pixel correlation, NPCR, UACI, and PSNR show that the encrypted images feature uniformly distributed histograms, entropy values close to 8, and significantly reduced spatial correlation. In addition, the experimental results of NPCR and UACI are consistent with their theoretical expectations. Robustness tests indicate that image cropping generally causes noticeable quality degradation, while the proposed scheme maintains relatively good visual fidelity under salt-and-pepper noise. Overall, the multi-dimensional validation—covering dynamic analysis, circuit implementation, and security performance evaluation—confirms that the proposed method can effectively resist statistical attacks, greatly increase the difficulty of plaintext recovery from cipher images, and provide an integrated and practical solution for secure RGB image encryption.

## Figures and Tables

**Table 1:** DNA Base Coding Rules Table.

Number	00	01	10	11
0	A	T	C	G
1	A	T	G	C
2	A	C	T	G
3	A	C	G	T
4	A	G	T	C
5	A	G	C	T
6	T	A	C	G
7	T	A	G	C

**Table 2:** Correlation coefficients of the image in the horizontal, vertical, and diagonal directions.

Test Diagram	Layers	Original Image			Encrypted Image		
		Horizontal	Vertical	Diagonal	Horizontal	Vertical	Diagonal
Sea	R	0.9122	0.9054	0.8643	−0.0080	0.0178	0.0218
	G	0.9188	0.9083	0.8728	0.0014	0.0022	−0.0099
	B	0.9048	0.9001	0.8537	0.0031	0.0099	0.0051
Meteorology	R	0.9489	0.9738	0.9454	0.0174	0.0066	−0.0055
	G	0.9475	0.9732	0.9407	0.0134	−0.0132	0.0217
	B	0.9088	0.9506	0.9006	−0.0063	−0.0142	0.0254
Vegetation	R	0.8528	0.8702	0.7800	0.0091	0.0167	0.0023
	G	0.8345	0.8475	0.7576	−0.0021	0.0048	0.0048
	B	0.8436	0.8571	0.7705	−0.0007	−0.0154	0.0061
Land	R	0.9316	0.9359	0.9010	−0.0061	−0.0223	0.0126
	G	0.9140	0.9369	0.8791	−0.0235	0.0098	−0.0052
	B	0.9100	0.9250	0.8767	0.0015	0.0005	0.0106
pepper	R	0.9479	0.9697	0.9403	−0.0058	−0.0152	−0.0230
	G	0.9844	0.9862	0.9765	−0.0018	−0.0028	0.0063
	B	0.9678	0.9725	0.9547	0.0033	0.0156	−0.0017
baboon	R	0.9164	0.8809	0.8736	−0.0062	−0.0062	−0.0043
	G	0.8685	0.7675	0.7399	−0.0134	−0.0125	−0.0060
	B	0.9041	0.8628	0.8363	0.0138	0.0122	−0.0102
sailboat	R	0.9544	0.9582	0.9428	0.0156	−0.0070	−0.0185
	G	0.9707	0.9675	0.9546	−0.0023	0.0095	0.0020
	B	0.9637	0.9655	0.9440	−0.0031	0.0021	0.0139

**Table 3:** Image Information Entropy.

Images	Layers	PlainImage Entropy	CipherImage Entropy
Sea	R	6.949016	7.999310
	G	7.664345	7.999241
	B	7.757246	7.999282
Meteorology	R	7.087825	7.999314
	G	7.117343	7.999274
	B	6.586042	7.999305
Vegetation	R	7.544950	7.999353
	G	7.175594	7.999401
	B	6.993260	7.999358
Land	R	7.448044	7.999258
	G	7.351227	7.999222
	B	7.065369	7.999262
Pepper	R	7.338827	7.999309
	G	7.496253	7.999329
	B	7.058306	7.999206
Baboon	R	6.499819	7.999237
	G	6.444500	7.999249
	B	6.270902	7.999096
Sailboat	R	7.312387	7.999323
	G	7.646107	7.999186
	B	7.213727	7.999339
Ref. [27]	R	7.2682	7.9992
Ref. [28]	-	7.4455	7.9991
Ref. [29]	-	7.2361	7.9991
Ref. [30]	-	7.1428	7.1998
Ref. [31]	R	7.7067	7.9992
Ref. [32]	-	7.7319	7.9920
Ref. [32]. pepper	-	7.6698	7.9920
Ref. [33]. baboon	-	7.66549665	7.99893469
Ref. [33]. pepper	-	7.74964698	7.99910385
Ref. [34]. pepper	-	-	7.9989
Ref. [34]. baboon	-	-	7.9990
Ref. [35]. sailboat	R	7.7570	7.9992

**Table 4:** NPCR and UACI of the algorithm proposed in this paper.

Images	Layers	1 bit Change		2 bit Change	
		NPCR	UACI	NPCR	UACI
Sea	R	0.996159	0.334459	0.996231	0.334990
	G	0.996426	0.334436	0.996029	0.334548
	B	0.996159	0.334956	0.996006	0.334492
Meteorology	R	0.996269	0.334936	0.996185	0.334539
	G	0.995922	0.334796	0.996071	0.334580
	B	0.996143	0.334236	0.996136	0.334427
Vegetation	R	0.995888	0.334021	0.996078	0.334328
	G	0.996002	0.334709	0.996025	0.334193
	B	0.995968	0.334577	0.996002	0.334541
Land	R	0.996292	0.334018	0.996151	0.334805
	G	0.996376	0.334326	0.995934	0.333778
	B	0.996399	0.334402	0.996166	0.334525
Pepper	R	0.996220	0.333754	0.996082	0.334220
	G	0.996017	0.335551	0.996052	0.335558
	B	0.996078	0.334974	0.996071	0.335046
Baboon	R	0.996175	0.334353	0.995904	0.335250
	G	0.996046	0.334036	0.996062	0.333882
	B	0.996192	0.335212	0.996129	0.333640
Sailboat	R	0.996010	0.334288	0.995979	0.334640
	G	0.996231	0.334380	0.996204	0.334976
	B	0.996143	0.334963	0.996059	0.334914

**Table 5:** NPCR and UACI of Other Algorithms.

	NPCR	UACI
Ref. [36]	0.996146	0.335341
Ref. [37]	0.99608	0.343827
Ref. [38]	0.996103	0.334692
Ref. [39]	0.996154	0.311348
Ref. [40]. R	0.995895	0.292090
Ref. [41]	0.996326	0.334924
Ref. [42]	0.997223	0.408491
Ref. [43]. pepper.R	0.995865	0.335472
Ref. [43]. baboon.B	0.995785	0.334114
Ref. [32]. pepper.R	0.9937	0.3342
Ref. [34]. pepper.G	0.99901	0.33732
Ref. [34]. baboon.R	0.99653	0.31543
Ref. [35]. sailboat	0.995982	0.334524

**Table 6:** PSNR under Clipping Attack and Salt-and-Pepper Noise Attack.

Images	Layers	Cropping Attack	Salt and Pepper Noise Attack
		PSNR	PSNR
Sea	R	19.917710	31.220322
	G	20.339068	31.248301
	B	19.819915	31.106182
Meteorology	R	19.176733	30.337105
	G	19.404439	30.183326
	B	20.880290	31.914344
Vegetation	R	19.925375	30.838594
	G	21.285003	32.048803
	B	19.074891	30.057004
Land	R	20.548157	31.410940
	G	20.656908	31.708639
	B	20.665007	31.723709
Pepper	R	20.576997	31.418529
	G	19.457361	30.829900
	B	19.668546	30.699319
Baboon	R	19.791639	31.446047
	G	19.989143	31.220487
	B	19.160160	30.695485
Sailboat	R	21.606305	32.079772
	G	19.278753	30.075009
	B	19.171763	30.257243

## Data Availability

The original data presented in the study are openly available in: www.srdatac.com (accessed on 15 February 2026); https://earthobservatory.nasa.gov/ (accessed on 15 February 2026); https://www.o-map.cn/demo/dth20200512-ndvi.html (accessed on 15 February 2026); https://www.sasclouds.com/english/home (accessed on 15 February 2026).
